# Prevalence of Chronic Kidney Disease and Progression of Disease Over Time among Patients Enrolled in the Houston West Nile Virus Cohort

**DOI:** 10.1371/journal.pone.0040374

**Published:** 2012-07-06

**Authors:** Melissa S. Nolan, Amber S. Podoll, Anne M. Hause, Katherine M. Akers, Kevin W. Finkel, Kristy O. Murray

**Affiliations:** 1 Department of Pediatrics, Baylor College of Medicine, Houston, Texas, United States of America; 2 Department of Internal Medicine, Medical School, University of Texas Health Science Center at Houston, Houston, Texas, United States of America; 3 Department of Epidemiology, School of Public Health, University of Texas Health Science Center at Houston, Houston, Texas, United States of America; University of Texas Medical Branch, United States of America

## Abstract

**Introduction:**

In experimental models of West Nile virus (WNV) infection, animals develop chronic kidney infection with histopathological changes in the kidney up to 8-months post-infection. However, the long term pathologic effects of acute infection in humans are largely unknown. The purpose of this study was to assess renal outcomes following WNV infection, specifically the development of chronic kidney disease (CKD).

**Methods:**

In a cohort of 139 study participants with a previous diagnosis of WNV infection, we investigated the prevalence of CKD using the Kidney Disease Outcomes Quality Initiative (KDOQI) criteria based on the Modification of Diet in Renal Disease (MDRD) formula and urinary abnormalities, and assessed various risk factors and biomarkers.

**Results:**

Study participants were primarily male (60%) and non-Hispanic white (86%) with a mean age of 57 years. Most (83%) were four to nine years post-infection at the time of this study. Based on the KDOQI definition, 40% of participants had evidence of CKD, with 10% having Stage III or greater and 30% having Stage I–II. By urinary dipstick testing, 26% of patients had proteinuria and 23% had hematuria. Plasma NGAL levels were elevated in 14% of participants while MCP-1 levels were increased in 12%. Over 1.5 years, the average change in eGFR was −3.71 mL/min/1.73 m^2^. Only a history of Neuroinvasive WNV disease was independently associated with CKD following multivariate analysis.

**Discussion:**

We found a high prevalence of CKD after long term follow-up in a cohort of participants previously infected with WNV. The majority of those with CKD are in Stage I-II indicating early stages of renal disease. Traditional risk factors were not associated with the presence of CKD in this population. Therefore, clinicians should regularly evaluate all patients with a history of WNV for evidence of CKD.

## Introduction

West Nile virus (WNV), a member of the Flaviviridae family, has become endemic in the United States, with more than 1.7 million persons estimated to have been infected [1]. The long-term effects of acute infection with this disease are largely unknown. Several animal models have been developed to understand the chronic pathologic effects of WNV infection. One model indicates that renal disease can result from acute experimental infection with WNV [2]. Importantly, after clinical recovery from WNV infection, animals develop chronic kidney infection with histopathological changes in renal tissue and viuria detectable for up to 8 months after acute infection.

Similar to the animal models, three studies have found evidence of virus in the urine of human WNV patients; however, these studies did not assess the renal impact of WNV infection [3], [4], [5]. In Houston, we previously reported that 9% of hospitalized WNV patients developed acute renal failure at the time of initial infection [6]. In this same cohort, only 8% of patients had a history of renal insufficiency prior to infection [7]. In Colorado a recent study assessing delayed mortality several years post infection, found that 21% of deceased WNV patients had documented renal failure as a contributory cause or underlying condition [8].

Thus far, the relationship between WNV infection and possible development of CKD in humans has not been thoroughly examined. We performed an observational study to determine the prevalence of CKD and examine biomarkers for renal dysfunction in a cohort of WNV study participants years past their initial infection.

## Methods

In 2003, a cohort of WNV patients was established in Houston, Texas and followed prospectively from the time of acute infection to up to nine years post-infection. To date, 217 patients have been enrolled into the study. Over time, 45 participants were withdrawn from the study either due to death, lost-to-follow-up, or voluntary withdrawal. Of the remaining eligible study participants, 139 had complete information to analyze for CKD prevalence. All study activities were approved by the University's Institutional Review Board (HSC-SPH-03-039) prior to subject enrollment.

For the purposes of this study, participants were assessed during April 2010-November 2011 and were evaluated every six months from the initial start date. Evaluation included a questionnaire and sample collections, including blood and urine. The questionnaire obtained the following demographic data: age, race, ethnicity, and height and weight for body mass index calculation. In addition subjects self-reported a diagnosis of diabetes mellitus, hypertension, or a family history of CKD, which are known risk factors for CKD.

Blood was analyzed by Quest Diagnostic Laboratories Inc. for complete metabolic profiles and complete blood counts [9]. Serum creatinine levels were obtained and estimated glomerular filtration rate (eGFR) was calculated using the Modification of Diet in Renal Disease (MDRD) study formula [10]. Chronic kidney disease was defined according to Kidney Disease Outcomes Quality Initiative (KDOQI) criteria for stages of CKD [11]. Participants were considered to have proteinuria and hematuria if urinalysis indicated greater than trace levels. Urinalysis was performed using Siemens Multistix® 10 SG reagent strips. Two potential biomarkers for renal dysfunction were also analyzed on a sub-set of fifty randomly selected participants. Neutrophil gelatinase associated lipocalin (NGAL) and monocyte chemotactic protein-1 (MCP-1) were measured in both plasma and urine using Quantikine Immunoassays (R&D Systems: Minneapolis, MN). We recorded the presence of common complications of CKD including anemia, elevated serum blood urea nitrogen (BUN), and hyperkalemia.

Descriptive statistics were used to describe the cohort and CKD risk factors, indicators and biomarkers. Univariate logistic regression analysis was performed to assess potential associations between stages of CKD with CKD risk factors. A cumulative grouping of all stages of CKD with CKD risk factors was subjected to multivariate logistic regression analysis to identify independent risk factors and test for potential confounding. All risk factors on univariate analysis with p≤0.25 were included in multivariate analysis. A backwards step elimination of the highest non-significant value method was used. Only those factors with p≤0.10 were considered significant in the multivariate analysis. All calculations were run using STATA® v12.0 software (College Station, TX).

## Results

A descriptive summary of the 139 participants is provided in Table 1. Timing of WNV infection in study participants ranged from recent (within the year) to up to 9 years, with the majority (83%) being more than four years post-infection. The population was predominately composed of non-Hispanic white males with a mean age of 57. Approximately one-half of the patients presented with an initial WNV diagnosis of acute Neuroinvasive WNV disease (meningitis and/or encephalitis). Mild (febrile) and asymptomatic infection comprised the other half of the population. Hypertension and advanced age were prevalent among the population.

**Table 1:** Descriptive summary of cohort demographics, CKD risk factors, CKD indicators, and CKD biomarkers.

	All WNV participants n = 139 (%)	Neuroinvasive WNV n = 67 (%)	Mild WNV n = 44 (%)	Asymptomatic WNV n = 28 (%)
*Years Post-Acute WNV Infection*				
Zero to Three	23 (17)	8 (12)	7 (16)	8 (33)
Four to Six	56 (40)	19 (28)	22 (50)	15 (26)
Seven to Nine	60 (43)	40 (60)	15 (34)	5 (8)
*Demographics*				
Male	84(60)	45(67)	22(50)	17(61)
Anglo	119(86)	52(78)	43(98)	24(86)
Black	9(6)	6(9)	1(2)	2(7)
Asian	2(1)	1(1)	0(0)	1(4)
Hispanic	9(6)	8(12)	0(0)	1(4)
*CKD Risk Factors*				
Aged 65 and older	42(30)	30(45)	9(20)	3(11)
Obesity	32(25)	18(28)	7(17)	7(25)
Diabetes mellitus	17(13)	14(22)	3(7)	0(0)
Hypertension	47(35)	29(44)	14(33)	4(14)
Family History of CKD	2(2)	1(2)	1(3)	0(0)
*CKD Prevalence*				
CKD, All Stages	55 (40)	32 (48)	12 (27)	11 (39)
CKD Stages 3–5	13 (10)	9 (13)	3 (7)	1 (4)
CKD Stage 1–2	42 (30)	23 (34)	9 (20)	10 (36)
*CKD Indicators*				
Proteinuria	36(26)	21(31)	9(20)	6(21)
Hematuria	32(23)	18(27)	7(16)	7(24)
Anemia	80(60)	40(60)	23(58)	17(63)
Elevated serum BUN	46(33)	30(45)	12(27)	5(18)
Hyperkalemia	16(12)	8(12)	7(16)	1(4)
Elevated plasma NGAL	7/50 (14)	5/24 (21)	2/16 (13)	0/10 (0)
Elevated plasma MCP-1	6/50 (12)	3/24 (13)	1/16 (6)	2/10 (20)

We found 40% of study participants met the KDOQI definition of CKD with 10% having Stage III or greater and 30% having Stage I–II. The prevalence of proteinuria and hematuria was 26% and 23%, respectively. Plasma NGAL levels were elevated in 14% of participants while MCP-1 levels were increased in 12%. Urinary levels NGAL and MCP-1 were elevated in 0% and 10%, respectively. The prevalence of anemia, elevated BUN, and hyperkalemia were 60%, 33%, and 12%, respectively.

**Figure 1:**
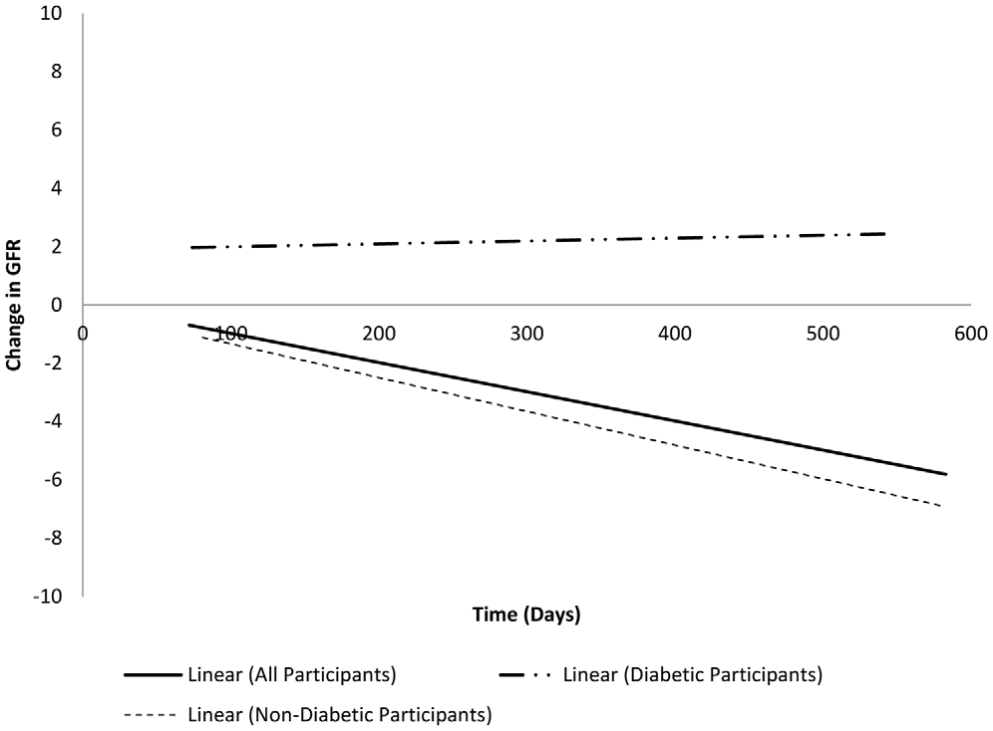
Change in eGFR values over time among 112 study participants, April 2010 to November 2011.

We analyzed the eGFR values and their change over the course of the study period (Figure 1). Follow-up data for analyzing the change in eGFR was available for 112 participants. The average change in eGFR was −3.71 mL/min/1.73 m^2^ over the 1.5 year study period. After adjusting for a leading cause of eGFR decline, non-diabetics had a significantly (p≤0.00) different decline in eGFR values than diabetics [12].

Results of the univariate logistic regression are provided in Table 2. Univariate analysis found Stage III or greater CKD was significantly associated with being age 65 or older (p≤0.01) and having hypertension (p = 0.01) at time of evaluation. No significant associations were found between Stage I–II CKD and CKD risk factors. Multivariate logistic regression was performed on the cumulative category of all stages of CKD with CKD risk factors. Based on univariate analysis, the following risk factors were included in the multivariate model: African American race (p = 0.10, OR = 3.31) and history of Neuroinvasive WNV disease (p = 0.06, OR = 1.95). After multivariate analysis, only Neuroinvasive WNV disease (p = 0.058, OR = 1.95) was found to be significantly associated with any stage of CKD.

**Table 2:** Univariate logistic regression analysis between presence of CKD and CKD risk factors and indicators.

	CKD, All Stages No. (%) p-value; OR (95%CI)	CKD, Stages 3–5 No. (%) p-value; OR (95%CI)	CKD, Stages 1–2 No. (%) p-value; OR (95%CI)
*CKD Risk Factors*			
African American Race	6/55 (11%) 0.101 3.31 (0.79–13.82)	1/13 (8%) 0.852 1.23 (0.14–10.68)	5/42 (12%) 0.086 3.31 (0.84–13.00)
Hispanic Ethnicity	4/55 (7%) 0.757 1.24 (0.32–4.83)	1/13 (8%) 0.852 1.23 (0.14–10.68)	3/42 (7%) 0.952 1.04 (0.26–4.25)
Aged 65 and older	18/55 (33%) 0.602 1.22 (0.58–2.54)	9/13 (69%) 0.004 6.34 (1.83–21.97)	9/42 (21%) 0.131 0.52 (0.22–1.21)
Obesity	12/54 (22%) 0.594 0.80 (0.35–1.82)	5/12 (42%) 0.160 2.41 (0.71–8.20)	7/42 (17%) 0.153 0.51 (0.20–1.29)
Diabetes mellitus	6/54 (11%) 0.672 0.80 (0.28–2.30)	2/13 (15%) 0.750 1.30 (0.26–6.43)	4/41 (10%) 0.579 0.72 (0.22–2.34)
Hypertension	22/55 (40%) 0.273 1.49 (0.73–3.06)	9/13 (69%) 0.011 5.03 (1.46–17.36)	13/42 (31%) 0.402 0.72 (0.33–1.55)
Neuroinvasive WNV Disease	32/55 (58%) 0.058 1.95 (0.98–3.88)	9/13 (69%) 0.122 2.64 (0.77–9.01)	23/42 (55%) 0.401 1.36 (0.66–2.80)

Family History of Chronic Kidney Disease was excluded from the table since there were insufficient numbers to calculate statistical values. OR = Odds Ratio, CI = Confidence Interval.

## Discussion

The goal of this study was to assess the long-term renal outcomes among a cohort of study participants with a history of WNV infection. In a previous study of WNV patients in Houston, we found 8% of participants had a history of renal insufficiency at the time of initial infection [7], and an additional 9% developed acute renal failure as a result of their illness [6]. In this follow-up study, we found 40% of our study population met the KDOQI definition for CKD when assessed up to nine years post-WNV infection. The majority of patients had CKD Stages I–II (30%) with CKD Stages III or greater being less frequent (10%). In multivariate analysis, traditional risk factors for CKD including age, and the presence of hypertension or diabetes mellitus were not associated with the presence of CKD. In fact, the analysis found history of Neuroinvasive WNV disease as the only significant risk factor associated with CKD. We cannot exclude severe infection with WNV as a factor in the development of CKD, especially considering the data from animal studies and the presence of persistent viuria in some human subjects.

Since CKD is a slowly progressive disease, we looked for any potential associations between years post-acute infection (PAI) and CKD. Due to sample size restraints, we grouped years into categories. We found an overall trend of increasing odds of CKD Stage progression as the years PAI increased. Those participants who were nine to seven years PAI had 1.61 greater odds than those with zero to six years PAI of having CKD Stages III–V, while those zero to three years PAI were more likely to have CKD Stages I–II. While a larger sample size is needed to confirm this finding, our data suggests that CKD progresses in the years following acute WNV infection. Chronic renal decline is also supported by findings of extended IgM titers several years post-infection in a substantial percent of WNV infected persons, including up to eight years post-infection [3], [13]–[15].

A previous study within this cohort found 20% (5/25) positive for viuria [19]. Viuria testing was not a main focus of this article since the authors are in the process of optimizing the viuria detection technique, and not all participants were tested for viuria due to logistic challenges. However, preliminary data does infer viuria testing as a possible diagnostic tool for WNV-related CKD. Of those who met the clinical definition for CKD, 50% (25/50) tested positive for viral RNA in the urine. In fact, more pronounced kidney decline was even more associated with viruira, with 83% (10/12) of those with moderate to severe CKD testing positive for viuria. While these findings are preliminary, we do intend to further investigate the possibility of viuria serving as a diagnostic marker for WNV-related renal disease.

Our data also suggest that WNV patients who are hypertensive or are 65 years or older are more likely to progress into stage III–V advanced renal disease. Since hypertension and older age are significant risk factors for severe WNV disease, it is unclear what their direct association is with the progression of CKD in this particular population. It is possible that these comorbidities serve a surrogate indicator of neuroinvasive disease's effect on renal decline. Current studies are underway aiming to evaluate long-term neurologic effects of WNV years post-infection and any correlation to renal decline among this population.

Evaluation of renal tubular injury biomarkers, NGAL and MCP-1, were not prevalent among our cohort. Increased NGAL levels have been demonstrated in several studies of kidney injury and we were not able to reproduce those findings [16]–[19]. MCP-1 is an indicator of interstitial inflammation among lupus patients [16]. Its low prevalence and lack of statistical significance suggests that MCP-1 is not relevant among our cohort and interstitial inflammation is not a dominant factor among our cohort. A larger sample size is needed to validate our findings.

We found a significant decline of eGFR values in our study population. Healthy populations have an average eGFR value decline of −1 mL/min/1.73 m^2^ per year [9], [10]. Our study population had an average eGFR value decline of −3.71 mL/min/1.73 m^2^ over 1.5 years. Even after adjusting for diabetes a known cause of eGFR decline [12], we still found the decline to be greater than expected. Additionally we found 15% of the study population had eGFR declines consistent with progression of CKD from milder to more severe stages over the 1.5 year period of observation. The lack of diabetes as an associated risk factor for eGFR decline suggests an alternate cause of progression. Therefore WNV may play a role in chronic renal dysfunction as noted by declining eGFR values.

We found a high prevalence of CKD among the cohort. While an alternative equation has been indicated as a more accurate estimator of eGFR values in the <90 range [20], we used the standard discipline for calculating our eGFR values. It is possible that using the standard equation could lead to inaccuracy and thereby misclassification. We ultimately decided to use the MDRD equation since it is believed to be the most commonly used equation by clinicians at this time. Additionally, since we used the KDOQI definition for stages of CKD which incorporates both eGFR values and evidence of kidney damage, we feel the specific equation used has minimal influence on the overall rates of CKD. When calculating the stages of CKD, we defined kidney damage as having proteinuria and/or hematuria. Proteinuria is known to be accurately measured by dipstick, although studies are inconclusive regarding the accuracy of dipstick measurement of hematuria [21]. Due to the observational nature of this study, we found dipstick measurements to be appropriate. Based on all the measurements we used to define CKD, we feel confident that our estimation that 40% of participants with CKD is accurate.

Based on our observational findings, it is evident that more extensive research is needed to understand the exact association between WNV and the development of CKD. We are in the process of developing an age-, gender-, and race/ethnicity-matched case control study with two control populations (CKD non-WNV patients and general medicine patients) to better understand the potential role of WNV infection and development of CKD. The main goals of this case-control study are to first identify if neuroinvasive WNV is serving as a surrogate marker for immunosuppression leading to renal decline. While our data does not suggest this is the case, we cannot rule it out without a proper control group. We anticipate to find that WNV is indeed a factor in renal decline, at which point we aim to then differentiate between factors associated with persistent WNV related renal decline and other causes of renal decline.

We found a high prevalence of CKD in long term follow-up of patients with a history of acute WNV infection. Over 1.5 years of follow-up, the average decline in eGFR was −3.71 mL/min/1.73 m^2^. The majority of those with CKD were found to be classified as Stage I–II, indicating mild disease. Those in this range are likely to be unaware that they are in the early stages of CKD and therefore at higher risk for disease progression. Traditional risk factors, such as diabetes, hypertension, obesity, and family history of CKD, were not found to be significantly associated with CKD among this population. However, there was an association with Neuroinvasive WNV infection at initial presentation. In light of our findings, we cannot rule out the influence of WNV with declining renal functioning. Physicians should monitor the kidney health of patients with a history of WNV infection.
